# Nitrofurantoin and glucose-6-phosphate dehydrogenase deficiency: a safety review

**DOI:** 10.1093/jacamr/dlac045

**Published:** 2022-05-03

**Authors:** Judith Recht, Vilada Chansamouth, Nicholas J. White, Elizabeth A. Ashley

**Affiliations:** 1 Mahidol-Oxford Tropical Medicine Research Unit, Faculty of Tropical Medicine, Mahidol University, Bangkok, Thailand; 2 Lao-Oxford-Mahosot Hospital-Wellcome Trust Research Unit (LOMWRU), Microbiology Laboratory, Mahosot Hospital, Vientiane, Lao PDR; 3 Centre for Tropical Medicine and Global Health, Nuffield Department of Medicine, University of Oxford, Oxford, UK

## Abstract

Nitrofurantoin, a broad-spectrum antibiotic available since 1953, is used widely for the treatment of urinary tract infections as it often retains activity against drug-resistant uropathogens. It is contraindicated in pregnant women at term, and in neonates. Like trimethoprim/sulfamethoxazole, nitrofurantoin carries a warning for patients with known sensitivity to oxidant drugs, notably glucose-6-phosphate dehydrogenase (G6PD) deficiency, in whom it may cause haemolytic anaemia. This is a barrier to uptake in tropical regions where there is a high burden of antimicrobial resistance and where G6PD deficiency is common. Early studies of erythrocyte survival following nitrofurantoin suggest it is less likely to cause oxidant haemolysis in individuals with G6PD deficiency than primaquine. Here we review reports of haemolysis associated with nitrofurantoin from the published literature and from USA (FDA Adverse Event Reporting System; FAERS) and European (VigiBase) pharmacovigilance databases. In total, 318 episodes of haemolytic anaemia were reported and 10 deaths, with 42 (13%) in individuals with confirmed or highly probable G6PD deficiency, out of at least 245 million exposures. A causal link between death and exposure was not reported and a precise risk estimation in G6PD-deficient individuals was not possible as there are few reports from regions where this enzymopathy is most prevalent. The evidence suggests a total daily dose of 200 mg nitrofurantoin may be used for short (3–5 day) course urinary tract infection treatment without G6PD screening when accompanied by appropriate advice. Pharmacovigilance in countries with high prevalence of G6PD-deficiency is recommended to monitor for serious adverse events.

## Introduction

Nitrofurantoin (NF; common brand names Macrobid®, Macrodantin®, Furadantin®) is a synthetic nitrofuran broad-spectrum antibiotic used for prophylaxis and treatment of uncomplicated lower urinary tract infections (UTIs) caused by common uropathogenic bacteria including *Escherichia coli, Enterococcus* spp. and *Klebsiella* spp. NF does not achieve sufficient tissue concentrations to treat systemic bacterial infections. Antimicrobial resistance is estimated to cause approximately 700* *000 deaths every year,^1,2^ with resistant Gram-negative infections on the rise globally. Available since 1953, NF is often active against ESBL-producing and carbapenem-resistant Enterobacterales, as well as vancomycin-resistant enterococci, and is recommended as a first-line treatment for uncomplicated UTIs (since 2010) by the Infectious Diseases Society of America, the European Society for Clinical Microbiology and Infectious Diseases^3^ and in Australia, where it has been available since the 1970s (NF reviewed in^4,5^). There has not been concerning development of resistance to NF, and cross-resistance with antibiotics and sulphonamides has not been observed.^6^ In a cross-sectional study in 2019 of 2848 urinary bacterial isolates from five European countries, the most common isolate was *E. coli* (2064) of which 98.5% were susceptible to NF including >90% of those isolates resistant to cefpodoxime (presumed ESBL producers or AmpC hyperproducers).^7^ One theory as to why nitrofurantoin resistance has not become widespread is that it confers a fitness cost to *E. coli*, such that resistant organisms are unable to establish an infection.^8^

### Pharmacokinetics

The only nitrofuran in human medical use, NF (C_8_H_6_N_4_O_5_, molecular weight 238.16 g/mol) is used widely in 3–7 day regimens as an oral antibiotic for UTI treatment. It is dispensed as 50 mg and 100 mg capsules; other formulations are a slow-release capsule and an oral suspension.^9^ Initial formulations were in microcrystalline form as NF monohydrate. This was replaced later by a macrocrystalline formulation with slower absorption leading to reduced frequency of administration and improved gastrointestinal tolerance.^10^ Oral NF is readily absorbed (40%–50% oral bioavailability; food increases absorption) and rapidly excreted, mostly in the urine. Therefore, NF accumulates in renal failure and should not be given to individuals with an estimated glomerular filtration rate (GFR) <30 mL/min^11^ or for long-term suppression, as recommended for older adults in the 2019 revised BEERS criteria of the American Geriatrics Society.^12^ In the UK it can be used with caution with estimated GFR in the 30–44 mL/min range to treat uncomplicated lower UTIs caused by suspected or proven multidrug-resistant bacteria if benefits are considered to outweigh risks.^13^ With normal renal function NF is eliminated rapidly (half-life 1–2 h) with maximum urinary excretion rates of approximately 10 mg/h. Peak plasma concentrations never exceed 2 and 4.6 μg/mL at therapeutic dosages under fasting and fed conditions, respectively. There is substantial inter-individual variability in plasma concentrations. No genetic polymorphisms affecting clearance have been identified.^14^

### Mechanism of action

The exact mechanism by which NF acts on bacterial UTIs is unknown. NF is reduced by several different enzymes, including bacterial flavoproteins, to reactive intermediates that interact with DNA and proteins. This results in inhibition of bacterial enzymes involved in carbohydrate synthesis at low concentrations, and inhibition of the synthesis of DNA, RNA, cell wall and proteins by action on bacterial ribosomal proteins at higher concentrations. Antibacterial activity is enhanced under acidic conditions.^9,15^ The multiple cellular targets may explain the lack of reported acquired bacterial resistance to NF.

### Current recommendations

The 2010 uncomplicated UTI treatment guidelines from the Infectious Diseases Society of America along with those of other US and European organizations, recommend the use of NF for acute uncomplicated cystitis as monohydrate/macrocrystals 100 mg twice (modified release; Macrobid®) or 50 mg four times (standard formulations) daily for 5 days.^3^ The UK National Institute for Clinical Excellence (NICE) guidelines recommend a 3 day course for children and adult women who are not pregnant, and 7 days for pregnant women and men with uncomplicated UTI.^13^

NF has been used extensively in longer regimens for treatment of UTIs, and for months or years prophylaxis against recurrent UTIs.^16^ In the USA, NF has been used since 1953, with more than 3 million prescriptions currently filled annually for UTI treatment, for which the recommended regimen is 50–100 mg orally twice daily for 5 days usually, and prophylaxis as 50–100 mg daily over the long term.^3^ Elsewhere 3 day regimens are usual, except for pregnant women, who are prescribed a 7 day course. The paediatric dose is 5–7 mg/kg daily.

### Adverse effects

Contraindications for NF use are significant impairment of renal function (different countries and recommendations use different cut-offs for creatinine clearance of <30 to <60 mL per min),^11,13,17,18^ and previous history of cholestatic jaundice/hepatic dysfunction associated with NF.

NF is also contraindicated in breastfeeding women and infants under 1 month of age, and its use is cautioned in people with anaemia, diabetes, electrolyte imbalances, folate or vitamin B deficiency, or any liver, nerve or lung disease. The most common reported side effects include nausea, vomiting, loss of appetite, and diarrhoea. NF is a potentially inappropriate medication in patients 65 years and older due to its potential for pulmonary toxicity, hepatotoxicity, and peripheral neuropathy when given long-term, e.g. as prophylaxis.^12^ Pulmonary toxicity is a severe reaction that can be acute, subacute, or chronic.^6^

NF is contraindicated in pregnant patients at term (38–42 weeks gestation), during labour and delivery, when the onset of labour is imminent, and in neonates less than 1 month of age.^18,19^ This is because of the possibility of haemolytic anaemia, the result of increased destruction of red blood cells (RBCs) and consequent RBC reduced life-span, releasing haemoglobin, as well as neonatal jaundice resulting from undiagnosed glucose-6-phosphate dehydrogenase (G6PD) deficiency or immature erythrocyte enzyme systems in newborns. Because the newborn’s susceptibility to oxidants (G6PD and pyruvate kinase deficiencies) is usually not known, NF often carries a warning for patients with G6PD deficiency, in whom it may cause haemolytic anaemia.^6,20,21^

### G6PD deficiency

The *G6PD* gene, on the X chromosome, shows extensive polymorphism with nearly 200 genetic variants described usually resulting in enzymatic instability. G6PD deficiency is the most prevalent enzyme deficiency worldwide, with an estimated 500 million people affected (reviewed in^22–25^). G6PD is the first enzyme in the pentose phosphate pathway, which generates NADPH (from NADP), required for the production of reduced glutathione in RBCs, and it is essential for the function of catalase. These provide cellular protection from oxidative stress. The activity of G6PD decreases exponentially as RBCs age, with a half-life of normal enzyme of about 50 days. In a 1976 report, in which several drugs were tested for haemolytic effects in G6PD-deficient individuals from southern China, NF was shown to shorten RBC survival (assessed by the half-life of ^51^Cr-labelled RBCs) from 19–28 days before drug administration to 4–14 days in three individuals with Canton, B(−)Chinese and one untyped G6PD deficiency variants, respectively.^26^ Details on NF dose and duration were not provided. Primaquine, also tested in that study at a dose of 15–45 mg/day, resulted in a greater shortening of RBC survival to a half-life of 3–5 days. In the early 1960s, after NF was shown to inhibit erythrocytic glutathione reductase *in vitro*,^27^ levels of reduced glutathione (GSH) in erythrocytes of normal and ‘primaquine-sensitive’ (G6PD-deficient black American individuals with presumed A− variant) were measured after 14 days of NF administration at a high dose of 800 mg daily.^28^ GSH was shown to decrease only in the G6PD-deficient individuals to the same magnitude, and with the same time course observed after administration of 30 mg primaquine daily. However, NF-induced haemolysis was not as pronounced, with a haematocrit reduction of only about 20%–25% of that caused by primaquine.^28^ The ^51^Cr-labelling experiments, including transfusions between primaquine-sensitive and non-sensitive subjects, showed that haemolytic anaemia after primaquine administration was self-limited: older RBCs were haemolysed whereas the younger erythrocytes were resistant to destruction (reviewed in^29^).

### Implications for current practice

Although NF is used as first line therapy for uncomplicated UTIs in many countries in North America, Latin America, Europe and Australia, it is not used in some southeast Asian countries, for example Lao PDR, where it is not registered and where G6PD deficiency is common.^30^

The treatment of UTIs is becoming more difficult. The current focus on strengthening AMR surveillance is showing high rates of ESBL production in *E. coli* across the world.^31^ For example, in Lao PDR 3317 urine specimens from inpatients and outpatients at Mahosot Hospital, Vientiane during 2017 and 2018 were cultured. *E. coli* was isolated from 163 samples (5%). The majority of *E. coli* isolates were resistant to ampicillin (153/163; 94%), followed by ceftriaxone (90/122; 74%), co-trimoxazole (115/163; 71%) and ciprofloxacin (86/162; 53%). In contrast, these isolates were highly susceptible to NF, amikacin and meropenem [152/160 (95%), 121/122 (99.2%), and 61/62 (98.4%), respectively].^32^ Amikacin and meropenem both need to be given by injection, are relatively expensive, and are regarded as reserve antibiotics. The advantages, especially affordability and low levels of antimicrobial resistance (AMR) compared with other antibiotics used to treat UTIs, suggest that NF should be considered as a UTI treatment option provided its safety profile is acceptable. We conducted an NF safety review of all haemolytic anaemia reports available from a variety of sources since the 1950s, when it first became available, with an emphasis on those confirmed in G6PD-deficient individuals.

## Methods

Searches were conducted using the terms [nitrofurantoin or furadantin or macrodantin] AND [hemolytic OR haemolytic OR haemolysis OR hemolysis] OR [nitrofurantoin or furadantin] AND [G6PD OR glucose 6 phosphate dehydrogenase] in PubMed, Embase and Global Health databases. Articles in other languages for which English titles came up were also included. Articles resulting from these searches that met the inclusion criteria (description of haemolytic anaemia cases in individuals due or possibly due to NF intake) and relevant references cited in those articles were reviewed. Several older case reports were identified from references within reviews, a book chapter and other publications and databases.

Adverse event reports due to NF from American and European pharmacovigilance databases were included. The US FDA’s Adverse Event Reporting System (FAERS) Public Dashboard was searched for all haemolytic anaemia cases, the information in an excel table was downloaded and a summary of the cases was included in this review. The Uppsala Monitoring Centre (UMC) WHO global VigiBase was also contacted and a file with reports of haemolytic anaemia, haemolysis and methaemoglobinaemia cases associated with NF was obtained and included here after cases were reviewed.

We have not attempted to grade or order the quality of the evidence, which consists mainly of case reports, hence low-quality evidence, and this was not a meta-analysis as the data were not amenable for such evaluations.

## Results

From all sources searched (literature and pharmacovigilance databases), a total of 318 episodes of haemolytic anaemia associated with NF intake were reported, 42 in individuals who were confirmed as G6PD deficient or in whom G6PD deficiency was highly probable. There were 10 deaths reported as associated with haemolytic anaemia and NF intake, all reported only in pharmacovigilance databases.

### NF-associated haemolytic anaemia cases reported in the literature

A total of 230 articles were obtained from the following databases: 90 from PubMed, 98 from EMBASE, and 42 from Global Health Trials, resulting in a total of 109 after removing duplicates. After reviewing the abstract and/or full text for inclusion and exclusion criteria, a total of 16 publications were included for review. An additional 14 publications came from references within the first 16 publications and other sources, including two reviews and one book chapter, for a total of 30 publications (care reports, studies, reviews) that reported haemolytic anaemia.

All cases reported in the literature are presented in Table 1, in chronological order of publication, followed by a summary of all cases from the literature (Table 2). A narrative describing all these cases with additional details is available in the Supplementary data (available at *JAC-AMR* Online).

**Table 1.** Nitrofurantoin (NF)-associated haemolytic anaemia reports in the literature in chronological order of publication^a^

Study first author, year, reference	Type of study	Haemolytic anaemia cases	NF dose and duration	G6PD-deficient patients?	Comments
West 1956^62^	Case report	1 28 year-old black American obese male with diabetes.	150 mg four times daily (600 mg per day), received a total dose of 2.4 g; probably not an overdose because of obesity.	Not specified; probably A− G6PD variant.	Patient recovered after NF discontinuation.
Kimbro 1957^63^	Case reports	2 African American males (42 and 48 years old).	400 mg NF daily for 9 days in one patient.	Not specified; probably A− G6PD variant.	RBC GSH stability was affected by NF and acetylphenylhydrazine, tested separately *in vitro*; two subsequent NF challenges in the second patient (48 years old) resulted in less-severe haemolysis.
Levy 1958^64^	Case report	1 (severe) 10 months old male baby.	450 mg or 55.6 mg/kg daily (10-fold higher NF dose than recommended).	Not specified; baby of Iraqi origin, possibly Mediterranean G6PD variant.	Patient recovered after drug withdrawal and blood transfusion.
Best 1963^65^	Report of 1962 additions to the Registry on Blood Dyscrasias	3	Not specified	Not specified	No details provided except for domestic (USA) cases.
Garret 1963^66^	Case report	1 25 year-old Jamaican woman pregnant with twins.	400 mg/day for 7–8 days.	Yes	Patient, probably in late pregnancy, experienced haemoglobin drops after Gantrisin (sulfisoxazole) and later NF.
Powell 1963^28^	NF study in ‘primaquine sensitive’ individuals	2 ‘primaquine sensitive’ African American males.	800 mg daily for 14 days (at least 4-fold higher than recommended dose).	Yes, probably A− variant.	GSH levels fell slightly from 45 to 30 mg/dL; the haematocrit fall was 20%–25% of that following 30 mg daily primaquine in primaquine-sensitive African-American individuals.
Deveber 1964^67^	Case report	1	5 mg three times a day for 4 weeks at approximate dose of 4.8 mg/kg.	Not specified	Premature baby on NF treatment for a UTI for 4 weeks; recovered after NF discontinuation and folic acid therapy.
Jeannet 1964^68^	Case report	1 (severe) after 2 days of NF treatment; 33 year-old Iranian woman at 35 weeks pregnancy.	300 mg/day for 2 days.	Yes, probably G6PD Mediterranean variant, demonstrated heterozygote.	Patient recovered and delivered a healthy male baby.
Pritchard 1965^69^	Case report	1 Black American 22 year-old pregnant woman.	2 days of 100 mg q6h, followed by half the dose for 4 days (100 mg twice a day).	Yes, probably A− G6PD variant; methaemoglobin test, quantitative G6PD activity test and ^51^Cr RBC half-life measurements at different times were performed.	Patient recovered quickly after NF discontinuation and folic acid; delivered a healthy male baby who tested severely G6PD deficient.
Burka 1966^34^	Retrospective study on 1956–64 G6PD-deficient patients in a US hospital.	3 probably caused by NF and 2 others probably caused by NF plus sulfa drugs.	Not specified	Yes	No details on patients; of 45 probable drug-induced cases, 20 were associated with sulphonamides.
Hibbard 1967^36^	Study on the effect of acute pyelonephritis with four drugs including NF in 87 pregnant women.	2 black American pregnant patients with acute pyelonephritis.	The maximum administered was 540 mg intravenously, then 400 mg orally.	Yes, both probably A− G6PD variant.	Both patients recovered from haemolysis and hepatic reactions after drug therapy change.
Dausset 1967^70^	Review of drug-induced haemolysis.	2 cases due to NF reported among 14 drug-induced haemolytic anaemia cases in Sardinia.	Not specified	High probability (13 out of the 14 cases reported were G6PD deficient); possibly Mediterranean variant.	Cases were unpublished observations, no details provided on patients.
Tool 1968^71^	Neural effects NF study on 14 subjects.	1	100 mg four times daily for 2 weeks.	Yes, black American subject, probably A− G6PD variant.	No details provided.
Maszkiewicz 1969^72^	Case report.	1 neonate.	The mother received methylene blue and NF starting on gestation week 32 until delivery.	Not tested	Haemolytic anaemia and methaemoglobinaemia in a preterm neonate who recovered.
Mital 1969^73^	One sentence in discussion section of a report on glutathione stability test of 323 people.	2 (mentioned in discussion briefly as unpublished data) NF-induced haemolysis in the 1966–68 period.	Not specified	Not specified	No details provided.
Steinberg 1970^74^	Case report	1 female	5 days	G6PD normal; patient (and 4 children tested) was deficient in glutathione peroxidase (GSH-Px); her activity levels were in the heterozygous range.	Patient received sulfisoxazole prior to NF.
Carpel 1970^75^	Case report	1 diabetic 83 year-old Italian male.	100 mg four times a day for 8 days (haemolysis occurred at 4 days).	G6PD activity test was within normal range.	Patient recovered after NF discontinuation.
Koch-Weser 1971^76^	Prospective study on AEs of three drugs including NF.	1	Not specified	Not specified	757 courses of NF treatment administered.
Stefanini 1972^77^	Case report	1 41 year-old female.	100 mg four times daily for 3 days.	G6PD normal; patient and sister were deficient in erythrocyte enolase.	Recovered after NF discontinuation, prednisone and blood transfusions.
Caldwell 1974^78^	A prospective study (1969-1972) at a USA hospital on antimicrobials adverse effects.	2	Not specified	Not specified	No details provided.
Herman 1975^37^	Retrospective study of haemolytic episodes in 129 G6PD deficient Kurdish patients in Israel.	3 pregnant women.	Not specified	Yes, measured by the Oski and Growney method; likely Mediterranean G6PD variant.	One patient also received streptomycin.
Lavelle 1976^79^	Case report	1 69 year-old African-American male.	100 mg three times a day for persistent pyuria for 4 days.	G6PD deficient, possibly A− variant.	Patient had undergone colon adenocarcinoma resection the same year; had metabolic acidosis and haemolytic anaemia; recovered in hospital.
Chan 1976^26^	^51^Cr RBC survival after 7–10 days drug administration in G6PD deficiency.	3	Not specified	Yes (1 Canton, 1 B-Chinese, one untyped).	NF shortened half-life of RBCs to 4–14 days, compared with primaquine reduction to 3 days after daily 30 mg dose.
Swanson & Cook 1977^35^	An NF chapter in book summarizing blood abnormalities associated with different drugs.	42	All cases were 200–800 mg/day for 4 days to 2 weeks duration.	22	All patients recovered after drug discontinuation. Several cases had been described.^34,36,37,62–64,67,69,74,75,77^
D’Arcy 1985^33^	Independent review of major reactions to NF based on Norwich Eaton Pharmaceuticals database.	42; 20 cases were G6PD normal: see Stefanini 1972^77^ for enolase deficiency, and Steinberg 1970^74^ for glutathione peroxidase deficiency.	All cases were 200–800 mg/day for 4 days to 2 weeks duration.	22	Haemolysis ceased upon drug withdrawal.
Gait 1990^21^	Review of NF haemolytic reactions based on Norwich Eaton Pharmaceuticals database.	127 reports of ‘haemolytic anaemia’ or ‘haemolytic anaemia as a result of G6PD deficiency’ ‘haemolysis,’ ‘primaquine-type reaction,’ and ‘Mediterranean phenomenon’.	No details	5 G6PD deficient; no record of G6PD testing for 102 patients. 5 patients (not tested for G6PD) had also received a sulphonamide.	Based on 127 global reports and 130 million courses in the USA alone, estimated risk of 1 haemolytic AE in 1* *023* *622 NF exposures (∼1 per million). 2 deaths (myocardial infarction and thrombotic thrombocytopenic purpura) not associated with haemolysis.
Bruel 2000^80^	Case report	1 male neonate.	Mother received NF in late pregnancy for 3 weeks before delivery.	G6PD normal neonate inferred from parents G6PD testing (normal).	Case considered a secondary haemolytic anaemia in the neonate due to maternal NF intake in late pregnancy.
Ghimire 2013^40^	Case report	1 71 year-old woman of northern European descent.	Not specified; patient also received phenazopyridine.	Diagnosed by G6PD testing.	Patient had two episodes of haemolytic anaemia after NF treatment for a UTI; the second time (this report) she was also on phenazopyridine for dysuria.
van de Mheen 2014^39^	Case report	1 28 year-old black Dutch woman, 33 weeks pregnant.	3 weeks of unspecified dose.	Yes, patient had haemolytic anaemia, hypertension and proteinuria and had received NF at 29 weeks.	Patient needed blood transfusions; responded to vit B12 and folic acid; also had α-thalassaemia (α,−3.7/αα) and sickle cell trait (HbAS).
Nasir 2014^81^	Case report	1 69 year-old Hispanic female.	NF and phenazopyridine (8 days each, doses not specified); phenazopyridine OD (8 instead of 2–3 days).	Yes	Patient recovered after blood transfusion.

AE, adverse event; OD, overdose.

a Haemolytic anaemia cases found in reviews include some of the case reports; when confirmed these are discussed in the narrative.

**Table 2.** Summary of nitrofurantoin (NF)-associated haemolytic anaemia reports in the literature

Case reports				Reviews		Total cases
Total cases	Cases in pregnant women and infants	G6PD deficiency cases	Possible NF OD^a^ and contributing drugs	Total cases after subtracting identified case report duplicates	G6PD deficiency cases	
**41** (37 before 1976 and before reviews)	8 pregnant women (all confirmed or probably G6PD deficient). 3 infants (one overdose in a possible Mediterranean variant; the other two secondary to maternal drug intake).	**24** confimed **4** highly probable	7 possible OD. 1 phenazopyridine OD. 1 (neonate); mother also received methylene blue. 2 probably due to NF plus sulfa drugs.	**22 new cases.** ^33,35,b^ **123 new cases.** ^21,c^ **145 total** ^d^	**5 new cases** ^33,e^ **5** ^21^ **10 total**	**186**

OD, overdose.

a Doses over 200 mg daily 5–7 days (uncomplicated UTI).

b A total of 42 cases reported in reviews in 1977^35^ and 1985,^33^ of which 20 were literature case reports.

c 127 in 1990;^21^ 11 pregnant women and 9 neonates (4 literature case reports).

d Subtraction of only confirmed haemolysis cases identified from references in the reviews corresponding to previously published case reports, this total is a conservative and probable overestimation of total cases, especially considering the second review in 1990,^21^ which probably included cases from prior publications.

e Total of 18 cases, including 9 confirmed and all 4 highly probable case reports.

We found a total of 41 cases of haemolytic anaemia episodes associated with NF reported in the scientific literature, including 17 case reports, of which 24 and 4 were in confirmed and highly probable G6PD-deficient patients, respectively. Eight and 13 cases, respectively, were reported in males and females, with gender not stated in 20 cases. There were eight cases in pregnant women, all confirmed or highly probable G6PD deficient, and three in infants (two cases due to maternal drug intake and one overdose), with eight possible overdoses including one in a neonate whose mother also received methylene blue, one phenazopyridine overdose, and two cases probably due to NF plus sulfa drugs.

Two reviews of global cases reported from a variety of sources to the NF developer Norwich Eaton Pharmaceuticals^21,33^ included 127 reports of haemolytic anaemia by 1990. Among these, five were in G6PD-deficient patients while there was no record of G6PD testing for 102 patients including five who had also received a sulphonamide. Two deaths (myocardial infarction and thrombotic thrombocytopenic purpura) were considered not associated with haemolysis; no additional deaths were reported. The use of concomitant drugs was often reported, including sulphonamides, which are included in a category to avoid in G6PD deficiency,^20^ such as in a retrospective study of haemolytic anaemia associated with G6PD deficiency from one centre in New York City (USA) between 1956 and 1964.^34^ Twenty out of 45 probable drug-induced cases of haemolytic anaemia in this study were associated with sulphonamides (sulfadiazine, sulfamerazine, sulfamethazine, and sulfisoxazole); three cases were classified as probably due to NF exposure, with another two probably due to NF and sulfa drugs.

Several cases in reviews have already been reported elsewhere. For example, from a book chapter published in 1977 summarizing drug-associated blood dyscrasias^35^ that reported 42 cases of haemolytic anaemia following 200–800 mg/day NF use (22 in G6PD-deficient individuals), several cases had been reported earlier in the literature including two individuals with hepatotoxicity (see Supplementary data).^34,36,37^

A systematic review and meta-analysis published in 2015 of 27 controlled trials and 4807 patients who received NF found low rates of adverse effects, which were mild, reversible and predominantly gastrointestinal, with no cases of pulmonary fibrosis and hepatotoxicity; the authors commented on what they interpreted as a publication bias focused on NF hypersensitivity reactions.^38^ Citing a review by D’Arcy,^33^ they described very low frequencies estimated for pulmonary reactions (0.001%), hepatic toxicity (0.0003%) neurological events (0.0007%) and haematological events (0.0004%).

### Pharmacovigilance databases

Haemolytic anaemia cases with NF as a suspect drug reported to the US FDA FAERS database were a total of 46 out of 52 after removal of duplicates: one case related to G6PD deficiency, two cases had been reported in the literature, and there were five deaths. Cases reported to VigiBase were 88 in total after removal of two duplicates, with three G6PD deficiency-associated cases reported, five deaths and 14 cases including as suspect other well-known drugs associated with a haemolytic risk in G6PD deficiency.

#### FDA Adverse Event Reporting System (FAERS)

The US FDA FAERS was searched in July 2020 for haemolytic anaemia associated with all different compound names including NF in them, a total of nine [‘NF/NF monohydrate’; ‘Ascorbic acid/NF’; ‘Hydrocortisone/NF’; ‘NF sodium’; ‘NF’; ‘NF macrocrystals’; ‘NF monohydrate’; ‘NF monohydrate macrocystals’; NF monohydrate/macrocrystals (monohydrate/microcrystal)’] resulting in a total of 52 cases reported in the database between 1970 and 2015 (Table 3). However, among these 52 cases reported there were duplicates and one quadruplicate, corresponding to a case report in the literature in a G6PD-deficient 28 year-old Dutch black pregnant woman^39^ (see case reports and Table 1). One duplicate event was another case report in the literature which we had not found by our search protocol, therefore we added it^40^ (see case reports and Table 1).

**Table 3.** Reports of nitrofurantoin-associated haemolytic anaemia from FAERS (US FDA)

Total	Duplicates and case reports	Total after elimination of replicates and case reports	Confirmed G6PD deficiency^a^	Pregnant women/infants^a^	Cases with other suspect drugs received (n)	Deaths
52	3 duplicates 1 quadruplicate 2 literature case reports	44	1	2/3	3 concomitant phenazopyridine (1 suspect) 1 noroxin (norfloxacin) 1 glimepiride (sulfonylurea)	5^b^

a The case in a pregnant G6PD-deficient woman reported in the literature^39^ was not included, as it is included previously in literature case reports (Tables 1 and 2).

b There were 6 deaths reported, among whom there was one duplicate.

After removing replicates, the total number of haemolytic anaemia events associated with NF reported to FAERS was 46. For several cases there was either age, country and/or event date missing, which when present were helpful to identify potential duplicates. In particular no G6PD status for any patient was included except for one also reported as an individual case report^39^ and one case in a 24 year-old female in 2010 classified as ‘non-serious’ including the following reactions: bone marrow failure; G6PD deficiency; caesarean section; haemolytic anaemia; maternal exposure during pregnancy; hepatic steatosis; hydronephrosis. Eleven cases total were classified as ‘non-serious’.

There were three cases in pregnant women, one of which was a death in a 28 year-old. There were also three cases in infants: an 8 day-old neonate whose reaction was entered as due to maternal drug intake, a 57 day-old baby who died, and a third case in a 2 month-old. There was one case in a 2 year-old child, while all other cases with patient’s age known (27 cases) were in individuals in the 27–88 years age range.

A female of unspecified age with haemolytic anaemia occurring in December 1995 had received phenazopyridine (pyridium) in addition to NF, both in the list of drugs to avoid in G6PD deficiency^20^ and both included in the suspect product name and active ingredients categories. There were five deaths reported: two of unspecified sex including the infant mentioned above, and three females including the duplicate also mentioned above.

#### VigiBase

VigiBase, the WHO global database of individual case safety reports (ICSRs) that currently includes almost 23 million reports, was contacted enquiring about cases related to NF intake that included G6PD, haemolytic anaemia, haemolysis and methaemoglobinaemia cases. An Excel file was obtained with a total of 780 entries corresponding to a total of 90 reports (Table 4); in one case there were 64 entries for the same report ID.

**Table 4.** Reports of nitrofurantoin-associated haemolytic anaemia from VigiBase

Total reports	Duplicates	Total after elimination of duplicates	G6PD deficient	Pregnant women/infants	Cases with other suspect drugs received (*n*)	Deaths
90	1	88	3	4/2	1 dapsone	5
	1 rechallenge				2 glimepiride	
					3 norfloxacin	
					3 phenazopyridine	
					5 phenazopyridine concomitant including one that also received pyrazinamide, acetylcysteine, isoniazid, rifabutin, rifampicin, phenazopyridine	

We identified a duplicate corresponding to an 18–44 year-old female in the Americas, which was reported on the same day in 2018 for a patient that was taking several drugs including a few in the ‘suspect’ category in addition to NF: pyrazinamide, acetylcysteine, isoniazid, rifabutin, rifampicin. This patient had tuberculosis and HIV infections, and had also received phenazopyridine as a concomitant drug.

There were three cases reporting G6PD deficiency; one in a male ≥75 years of age in the Americas who recovered that was reported in 1968, a second one in the Americas in an 18–44 year-old female who had concomitant phenazopyridine reported in 1989, and a third one in Asia in an 18–44 year-old female reported in 2019. Several cases reported phenazopyridine as a suspect (*n *= 3) or concomitant (*n *= 5) drug, one such case in a female 45–64 year-old had received three suspect drugs (NF, phenazopyridine and sulfadiazine).

Another case had taken dapsone, which carries a well-known high risk of haemolysis in G6PD-deficient patients, and was included as a suspect drug in this 45–64 year-old female case reported in the Americas in 2008. Two other cases in ≥75 year-old females in Europe had received glimepiride for type 2 diabetes, a sulfonylurea drug associated with a haemolytic risk in G6PD-deficient patients and post-marketing reports of haemolytic anaemia in non-G6PD-deficient patients.^41^

There were five deaths from haemolytic anaemia reported with NF as a suspect drug; two in the Americas (reported in 1992 and 2005, respectively); two in Europe (one reported in a ≥75 year-old female in 2011 with circadin (melatonin) also as a suspect drug and several other concomitant drugs, and the second one in 2011 in a 45–64 year-old female); and one in Oceania in 1973.

### Haemolytic anaemia in patients treated with NF

#### Total haemolytic anaemia cases from all sources

All haemolytic anaemia cases reported from the literature and databases, including subsets of G6PD confirmed and highly probable cases, were added regardless of whether they were due to an overdose, maternal drug exposure or possible roles of suspect or concomitant drugs (Table 5). These estimates may be an overestimation of reported cases as there could still be unidentified duplicates between the databases and literature reports (there was very limited case information for some cases in databases), as well as cases possibly due to (or contributed to by) other suspect and concomitant drugs with well-known haemolytic risk, cases due to NF overdose, and those reported in infants due to maternal drug exposure. On the other hand, there are also probably cases that are not reported and therefore not captured in any of the sources included here.

**Table 5.** Reports of nitrofurantoin-associated haemolytic anaemia in the literature and databases

Characteristics	Cases reported in the literature	Cases summarized in Reviews	US FDA (FAERS) + VigiBase cases^a^	Conservative total after elimination of confirmed duplicates
Total cases	41	145	44 + 88	318
G6PDd confirmed	24	10	1 + 3	38
G6PDd confirmed + highly probable	28	10	1 + 3	42
Deaths	None	2 (considered not associated with haemolysis, may be duplicates/FAERS reported)	5 + 5	10

a From Tables 3 and 4, respectively.

#### Total NF course exposures estimate

Until 1990 there were an estimated 130 million courses of NF prescribed in the USA.^21^ Since then, the number of prescribed courses of NF in the USA has averaged 3 million annually,^42,43^ i.e. a total of approximately 220 million prescribed courses overall. For Canada, data reported per year for 2000–13 showed a clear continuous increasing trend from 14.61 to 34.61 NF prescriptions/1000 population.^44,45^ Using population data from Canadian official sources,^46^ we estimated about 10.24 million prescriptions for 2000–13, and used a conservative estimate of 1* *214* *222 annual prescriptions (total number of prescriptions for 2013) for subsequent years to result in approximately 19 million NF prescriptions in Canada for 2000–20.

In New Zealand, 283* *561 patients received NF from a community pharmacy between 2013 and 2017 according to the country’s Medicines Adverse Reactions Committee.^47^ In Australia, NF was reported in 2008 to have been widely available and used for over 30 years for UTI treatment and prophylaxis, with about 120* *000 prescriptions per year dispensed by the Pharmaceutical Benefits Scheme (PBS) alone.^48^ We used a conservative estimate for these two countries of 100* *000 prescriptions/year for the last 20 years, for a total of 4 million prescriptions for 2000–20.

A recent study of lower UTI antibiotic use in England in 300* *354 patients in 390 general practices in 2011–15 found that NF was the second most commonly prescribed antibiotic (23.9%) after trimethoprim (56.8%),^49^ and in 2019 it was also the second-most dispensed antibacterial drug after amoxicillin.^50^ There were 284* *264 NF prescriptions in England in 2013–15 according to primary care data recorded in The Health Improvement Network (THIN) database.^51^ In the Netherlands, 215* *531 NF prescriptions were reported for women from 1996 to 2015, a period in which NF was the most prescribed drug for UTI treatment in women.^47^ Using the same estimate as above of 100* *000 annual prescriptions, there were about 2 million NF prescriptions in England for 2000–20.

Together, estimates above from countries in different continents (USA, Canada, England, the Netherlands, New Zealand and Australia) for the periods for which data were available, add up to an estimated 245 million NF exposures. This is a considerable underestimation of NF prescriptions globally, as it is a widely prescribed antibiotic in other countries as well, including in Latin America, for which we found no NF prescriptions data.

#### Estimate of NF haemolytic anaemia risk

**Figure 1.**
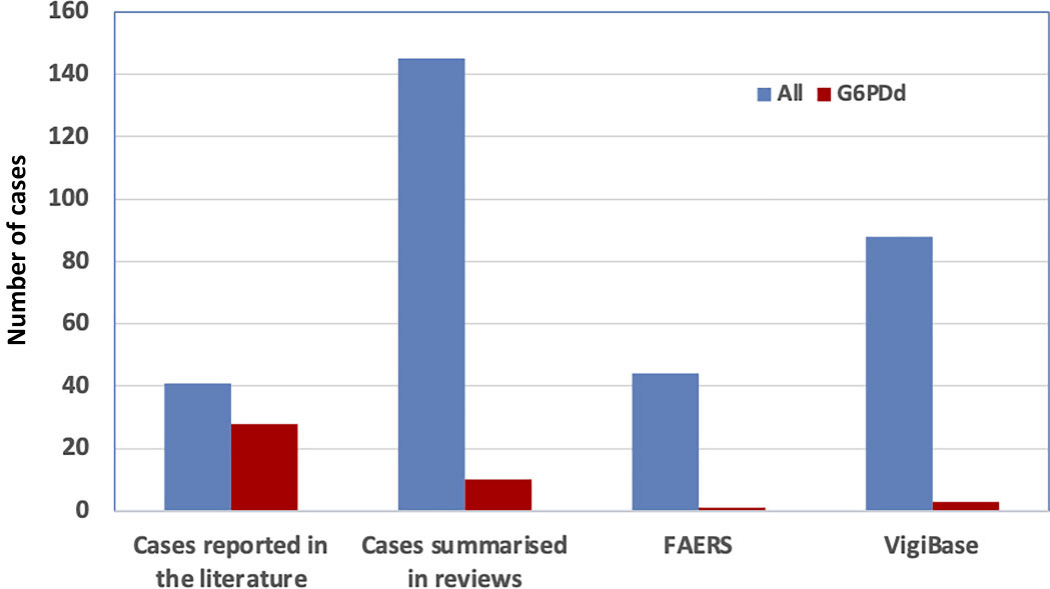
Nitrofurantoin-associated haemolytic anaemia cases by data source. Total number of cases reported in the literature, cases summarized in literature reviews (confirmed non-duplicates of case reports), cases in pharmacovigilance databases (US FDA/FAERS and European/WHO VigiBase) are shown by blue bars; cases in confirmed or highly probable G6PD-deficient individuals are shown by red bars.

Combining the number of reports with the number of exposures gives an estimate of 1.3 haemolytic anaemia cases per 1 million NF exposures (95% CI: 1.16–1.45 cases/million), and 1 death associated with haemolytic anaemia in 24.5 million exposures reported or 0.04 cases per million (95% CI: 0.022–0.075/million). However, such estimates have limited value since critical details to ascertain causality were often lacking, many cases had no information on G6PD testing (Figure 1), or the patient had not been tested, and most of the exposures were in populations generally considered to have a lower frequency of G6PD deficiency.^21^

Primaquine has been used as an antimalarial since the 1950s. The main adverse effect is haemolysis in G6PD deficiency. In a primaquine safety review published in 2014, we reported a total of 14 deaths associated with primaquine use worldwide, with an estimated risk of 1 in 621* *428 exposures.^52^ This makes the estimated risk of death associated with haemolytic anaemia for NF in this review about 40-fold lower than that of primaquine. However, primaquine is used in countries with higher prevalences of G6PD deficiency and the estimate is for all-cause mortality.

Males are hemizygous for G6PD deficiency, showing either normal G6PD activity levels or G6PD deficiency depending on the *G6PD* allele they carry (wild-type or *G6PD* variant). Homozygous-deficient females (with a frequency which is the square root of the prevalence in hemizygous males in a given population^53^) are as deficient as hemizygous males, while the heterozygous female population phenotypically shows a wide G6PD activity range from normal to deficient levels due to embryonic random X-chromosome inactivation in individual cells (Lyonization). Phenotypically, G6PD deficiency is most often seen and reported in hemizygous males, on whom most of the data and studies on G6PD prevalence are based.^54^ In 1989, the World Health Organization (WHO) reported G6PD deficiency prevalences of 0.5%–2.9% of hemizygous males in the USA, compared with average prevalences of 8%–10% in many malaria-endemic countries in sub-Saharan Africa, and in some areas prevalences up to 30%.^53^ Thus, in high-income countries including the US and many in Europe from where NF data and denominator estimates for risk calculations are derived, G6PD prevalence is generally much lower than in other countries. In addition, NF is predominantly prescribed to women for UTI treatment,^55^ as shown by a 2011–15 study in England of 494* *675 UTIs diagnosed in 300* *354 patients in which NF was the second most prescribed antibiotic and 83.3% of the patients were women.^49^

### Varying restrictions and recommendations on NF use

New restrictions on the use of NF were issued by Spain in 2016 that included treatment limited to a maximum of 7 days and informing patients about (non-haematological) pulmonary, hepatic, allergic and neurological risks.^56^ Although similar restrictions were issued by France, no alerts were detected from other international regulatory agencies from the USA, Europe (EMA), Argentina, Canada, Australia, Mexico, Brazil, Perú and the UK. A review of pharmacogenomic drug labels and regulations from the US FDA and the European Medicines Agency (EMA) during 2015–18 found that for NF, for patients with G6PD deficiency, the FDA had pharmacogenomic information only, whereas the EMA stated contraindications instead.^57^ A study on 2006 antibiotic prescriptions at a military hospital in Colombia’s capital Bogotá reported DDD of 9.5/1000 patients for the year for NF (equivalent to approximately 88* *000 courses of therapy) and showed that NF was the fourth-most prescribed antibiotic (5.4% of all antibiotics).^58^ As with the antimalarial primaquine, there is no requirement for G6PD testing prior to prescribing the drug, which may be avoided when G6PD deficiency is known in the patient, resulting in unknown number of exposures to NF in G6PD-deficient individuals.

### Limitations

Many cases in the different drug databases lacked details on one or more of the following: NF dose, age and gender of the patient, severity, outcome of the event and, importantly, G6PD status. We were able to identify duplicate events within each database and between sources (literature and databases; case reports and reviews) when the information included permitted it. However, there may have been additional duplicates not clearly identifiable based on the case information provided. We leaned towards a conservative estimation of risk in all considerations, thus we included all haemolytic anaemias from all sources, even those that may have resulted from other suspect or concomitant drugs or underlying disease. There were cases due to NF overdose, as well as cases in infants due to maternal drug exposure, which would not occur based on current recommended NF dosing and contraindications. Of note there were no case reports of deaths from haemolytic anaemia following NF treatment in the literature, while there are reports of deaths from other complications such as hepatic necrosis.^59^

A NF review of adverse events published in 1985 (see reviews and Table 1) calculated occurrence rates of pulmonary and neurological reactions at 10 and 7 per million courses of therapy, respectively,^33^ higher than the risk estimated here of 0.17 per million for haemolytic anaemias. However, haemolytic anaemia risk is relevant mainly for a subset of the population, G6PD-deficient individuals,^60^ and the lack of NF studies that specifically included G6PD-deficient patients in the literature as well as most reports available being from populations with low prevalence of G6PD deficiency made it impossible to calculate the incidence and estimate the risk of haemolytic anaemia events in this population. The fact that women receive the majority of antibiotic courses for UTI will also dilute the assessment of risk, since women with G6PD deficiency are usually heterozygous, with a phenotypic range of G6PD activity including higher levels than hemizygous males.

### Proposed recommendation for countries planning to introduce NF to their formularies

In many countries NF has been used extensively for several decades, with a total daily dose of 200 mg recommended for uncomplicated UTIs. From the 318 cases of haemolytic anaemias reviewed here described in the literature with details on dose and duration, four cases before 1963 (more than half of those with dose details) were associated with higher than the 200 mg daily for 5–7 days dose recommended currently for uncomplicated UTIs or the 3 day regimen used in many countries including the UK. The WHO Essential Medicines List Antibiotic Book due to be published in 2022 will recommend a 5 day regimen.^61^ Recommending NF in countries where G6PD deficiency is common should therefore start with the lowest dose and duration regimen (200 mg daily for 5 days). NF should be contraindicated in late pregnancy and in patients with documented or self-reported haemolytic reactions to other medicines, and be accompanied with warning patients about possible side effects and haemolytic anaemia signs as well as instructions to stop taking NF if these signs, including dark urine, occur.

## Conclusions

NF is a drug of choice for empiric UTI treatment and prevention as it is associated with lower bacterial resistance compared with other antibiotics prescribed for this condition and lower propensity to select for resistance. Increasingly, it may be the only oral agent able to treat uncomplicated UTIs caused by ESBL-producing Enterobacterales. It is also inexpensive and generally well tolerated. Although it is an oxidant drug, early studies of erythrocyte survival following NF suggest it is less likely to cause oxidant haemolysis in individuals with G6PD deficiency than primaquine. This is supported by the low number of reported adverse events following NF intake reviewed here. Furthermore, the short course of treatment limits the anaemia if there is oxidant haemolysis. Ten deaths have been reported to be associated with haemolytic anaemia and NF treatment, however a causal relationship was not confirmed and other concomitant drugs were associated in some cases.

Provided that advice is given to stop the drug if signs or symptoms of significant haemolysis occur, fatal haemolytic anaemia should be completely avoidable. The accumulated evidence suggests that NF may be used for short course treatment of UTI without screening for G6PD deficiency, accompanied by appropriate advice. A similar approach is taken for trimethoprim/sulfamethoxazole, which is a very commonly prescribed antimicrobial drug in similar settings. However, because most published data are from countries with a low prevalence of G6PD deficiency, controlled studies in patients with known G6PD deficiency, including pregnant women, would be useful, particularly in countries with higher prevalence and different variants and severity.

In summary, although NF can cause haemolysis in G6PD-deficient patients, the totality of evidence accumulated over nearly 70 years suggests that this risk is low with currently recommended regimens and that NF can be prescribed without testing.

## Supplementary Material

dlac045_Supplementary_Data

## Supplementary data

More details on the cases of haemolytic anaemia are available as Supplementary data at *JAC* Online.
