# Supplementary material for: Nitrofurantoin and glucose-6-phosphate dehydrogenase deficiency: a safety review
Source: JAC Antimicrob Resist. 2022 May 3;4(3):dlac045. doi: 10.1093/jacamr/dlac045 (PMC9070801; doi:10.1093/jacamr/dlac045)
Supplement: dlac045_Supplementary_Data [file dlac045_supplementary_data.docx]

### Supplementary data

Here we provide a more detailed narrative of all the haemolytic anaemia cases reported in the literature, including those summarised in Table 1 in the review.

### *Case reports*

The first case of haemolytic anaemia associated with NF treatment was reported in the literature in 1956. It described a 28-year-old African-American man with diabetes mellitus. On the fourth day of a hospital stay he was given NF at 150 mg four times a day and passed “coffee-coloured” urine and had a haemoglobin of 8.8 g/dL; he recovered after NF was discontinued when he had received a total dose of 2.4 g.^1^ Although the total daily dose (600 mg) given to this patient was higher than the currently recommended 200mg NF, he was an obese male who weighed 122.5 kg, thus we did not classify this case as an overdose. The authors state “If the anaemia is related to the drug, it must be an extremely rare occurrence, since no previous reports of this untoward reaction have appeared”. The patient was probably G6PD deficient (A- variant). The authors referred to an RBC abnormality analogous to primaquine sensitivity reported in healthy African American individuals.

Another two cases of haemolytic anaemia after NF intake in two black American males (42 and 48 years old, respectively) in the USA were reported in 1957 in hospitalized patients who received 400 mg daily NF as UTI treatment.^2^ The first patient received NF for 9 days, by which time his haematocrit had fallen from 41% to 27%, with a reticulocyte count of 6.5%; he recovered after NF discontinuation with a haematocrit back to 41% one month after he first received NF. The second patient also received streptomycin along with NF and developed fever, jaundice and tachycardia and a fall in haematocrit to 25.5% with a reticulocyte count of 2.4% on the fifth day, when he received a blood transfusion and recovered after 35 days from NF treatment initiation. These two individuals were studied further including *in vitro* measurement of RBC GSH stability to acetylphenylhydrazine and NF, upon either of which the GSH cell content fell drastically. The authors included GSH testing of samples from 296 black individuals for whom were classified as “reactors” with a higher incidence in males than females. The second male was re-challenged twice with NF for ten days each time, with a haemolytic response each time although much less intense than the first one, which the authors compared to primaquine-induced anaemia that had been recently studied in detail in African-American populations. These results at the time supported the existence of a common RBC abnormality for primaquine- and NF-induced haemolytic anaemias in what was subsequently shown to be G6PD deficiency. The decreased severity of subsequent haemolytic reactions is well-described in G6PD-deficient individuals who are repeatedly exposed to primaquine. It is explained by a brisk reticulocytosis after the initial insult with increased production of younger red blood cells which are more resistant to oxidant haemolysis.^3, 4^

In 1958, a case of severe haemolytic anaemia was reported in a 10 months old male baby of Iraqi origin weighing 8.1 kg associated with NF (Furadantin$®$). This was administered in Israel in hospital for a UTI the baby developed on the third day after he was admitted with severe metabolic acidosis.^5^ The infant received 50 mg NF three times daily, and passed dark red urine on the fourth day of NF treatment after a total dose of 700 mg (90 mg/kg). The haemoglobin fell to 3g/dL. At this point NF was discontinued and the patient received a blood transfusion. The haemoglobin recovered to 7.4 g/dL and a week later to 9.8 g/dL; the baby was discharged after 28 days in hospital. The daily dose administered to this patient, 450 mg per day or 55.6 mg/kg daily is 10 times higher than the currently recommended paediatric dose of 5-7 mg/kg daily and corresponds to an overdose in what was most likely to be a Mediterranean G6PD variant patient.

A case reported in 1963 described severe haemolysis in a 25-year-old G6PD deficient Jamaican woman pregnant with twins, which occurred twice in the same month after she received first Gantrisin$®$ (sulfisoxazole) for acute pyelitis for about 5 days and later NF (Furadantin$®$) for 7 days.^6^ Details of the pregnancy were not provided; the patient underwent surgical induction the day NF was discontinued, delivered twins that did not survive (one stillborn following prolapse of the cord and a breech) and left hospital on the tenth day with a haemoglobin of 10.8 g/dL (71%). G6PD deficiency was diagnosed based on two tests (brilliant-cresyl blue dye screening (Motulsky) and the method of Zinkham, Lenhard and Childs). Based on the haemoglobin levels provided in the report, the haemoglobin drop resulting from sulfisoxazole was at least 3-fold greater than the one observed with NF.

A haemolytic anaemia case was reported in 1964 from Canada in a prematurely born 2.5 months old baby who received NF treatment for a UTI at 5 mg three times a day for one week at the hospital and another 3 weeks after discharge.^7^ His weight on admission was 6 lb 14 oz (3.1 kg), with 7.6 g/dL haemoglobin and a reticulocyte count of 2.1%. Although he had been on 15 mg iron supplementation for a month, he was diagnosed with anaemia and the iron dose was increased to twice daily. When readmitted at the hospital a month later when he was pale, listless, irritable, and losing weight, his haemoglobin was 5-6 g/dL with a reticulocyte count of 3% and NF was discontinued. He received folic acid and recovered, with haemoglobin levels up to 10.6 g/dL two weeks later.

A case of severe haemolysis was reported in 1964 in Switzerland in a 33-year-old Iranian pregnant woman at 35 weeks gestation after NF treatment for a UTI.^8^ She was first treated with sulfisoxazole, followed by NF 300 mg/day, to which she developed a reaction evident after 2 days with jaundice, fatigue and red-brown urine, with 7.6 g/dL haemoglobin. She was given hydrocortisone, prednisone and a blood transfusion and in 2-3 days her haemoglobin levels recovered to 10.1 g/dL, followed by complete recovery from haemolysis in 4 more days, when she delivered a healthy male baby. This interesting paper highlights new (by then) assays to evaluate G6PD deficiency. The authors showed G6PD deficiency and its heterozyogosity in this patient by acetylphenylhydrazine staining of Heinz bodies (inclusion bodies in erythrocytes due to haemoglobin denaturation and binding to the cell membrane), and methaemoglobin reduction test ^9^ to reveal a mixture of normal and G6PD deficient RBCs, which they compared to a hemizygote (male) G6PD deficient patient’s film showing a homogeneous G6PD deficient population.

A case was reported in 1965 describing a black American 22-year-old woman in her fourth pregnancy who developed haemolytic anaemia with haemoglobin dropping from 12.7 g/dL to 6.9 g/dL and megaloblastic erythropoiesis after 6 days of NF.^10^ The patient reported that she had started taking NF 100 mg every six hours for two days and, after feeling weak, dizzy, anorexic, vomiting and with a headache that she attributed to the medication, she reduced the dose to twice a day. A methaemoglobin reduction test in the patient indicated a moderate degree of G6PD deficiency, while quantitative measurements with the modified Glock and McLean method showed 80 units of G6PD activity on admission (half of normal RBC activity), 40 units three weeks later and 22 units six months after delivery, in agreement with G6PD activity in G6PD deficiency being measured as higher (sometimes normal) after acute haemolysis because of the loss of haemolysed older RBC. This phenomenon was also shown in this patient by ^51^Cr RBCs half-life measurements: before NF intake 67% of labelled cells had disappeared after 13 days (equivalent to losing 50% of RBC activity in 11 days) compared to more than 30 days for normal pregnancies at this stage (this latter value is cited in this paper as Pritchard, unpublished data) while another RBC survival measurement of the patient in hospital was 36 days. The patient recovered quickly after NF was discontinued and she received folic acid; she delivered a male baby who tested as severely G6PD deficient by methaemoglobin reduction test and 9 units (very low) in a quantitative G6PD test in G6PD normal activity levels were around 160 units. However, in 1968 a report based on G6PD enzymatic activity measured from 100 pregnant women at the third, sixth, seventh, eighth and ninth months of gestation using Zinkham and Lenhard’s modified technique showed normal or increased activity in 75 % of women at the beginning of pregnancy, which declined in 65% of women to about half of the normal activity, returning to normal after childbirth.^11^ This observation has not been replicated in other published reports.

A case in a neonate suffering from haemolytic anaemia with methemoglobinemia who recovered was reported in 1969.^12^ The mother had been treated for a UTI with methylene blue (which is also associated with haemolysis in G6PD deficiency^13^) and NF starting on week 32 of pregnancy until premature delivery following antepartum haemorrhage; G6PD testing was not done on the mother or the neonate.

A case of haemolytic anaemia in the USA was reported in 1970, describing an 83-year old Italian male diabetic patient admitted to hospital in a semi-comatose state. He developed a urinary infection at the hospital and was given NF at 100 mg four times a day (along with insulin for high blood sugar) resulting in haemolysis at 4 days, which was discontinued after 8 days of treatment when haemoglobin fell to 5.9 g/dL, after which the patient recovered.^14^ Levels of G6PD were measured during haemolysis and one month later and were within the normal range.

In 1976 a case report was published describing a 69-year-old black male in the USA with less than 1 IU/g haemoglobin G6PD enzymatic activity (normal range 5-10 IU/g) following a colonic adenocarcinoma resection, who developed lactic acidosis and haemolytic anaemia (haemoglobin levels 8.6 g/dL) after 4 days of NF treatment of 100 mg three times a day for persistent pyuria.^15^ The metabolic acidosis subsided spontaneously within 2 days and the patient was discharged from hospital 21 days later from hospital.

Haemolytic anaemia due to maternal NF intake for 3 weeks on the ninth month of pregnancy until delivery was reported in 2000 in a full-term neonate whose parents were G6PD normal;^16^ the authors considered it secondary haemolytic anaemia due to the maternal drug.

A case reported in 2013 in the USA described fatigue, dyspnoea and dark-coloured urine in a 71-year-old woman of northern European descent who had been taking NF for UTI treatment and phenazopyridine for dysuria.^17^ Her haemoglobin level was 5.5 g/dL with reticulocytosis, raised indirect bilirubin and lactate dehydrogenase. Even though G6PD levels were normal, a peripheral blood smear showed features of G6PD deficiency; thus, both oxidant drugs were stopped and when G6PD levels were retested three months later they were low, confirming the G6PD deficiency diagnosis. The patient had previously had symptomatic anaemia after NF intake for a UTI that required a blood transfusion, with a two weeks later follow-up showing normal values including for G6PD. As pointed out by the authors of this case report, G6PD levels in blood can be within normal range after a recent blood transfusion or during acute haemolysis.

In 2014 a case was reported of a 28-year-old black Dutch pregnant woman admitted to hospital at a gestational age of 33 weeks with haemolytic anaemia, hypertension and proteinuria who had received NF at 29 weeks when she developed cystitis.^18^ When the patient presented to hospital 3 weeks later she needed blood transfusions. Molecular and chromatography analyses revealed coinheritance of α-thalassaemia (α,-3.7/αα) and sickle cell trait (HbAS), with G6PD screening revealing decreased G6PD activity in erythrocytes 1.7 IE/g Hb (reference range 3.8–5.9 IE/g Hb). Another case reported in 2014, as a conference abstract, described a 69 year old Hispanic female who had taken NF and phenazopyridine for 8 days who presented with shortness of breath, fatigue, and malaise and haemoglobin of 6.8 g/dL compared to 12 g/dL a few months earlier, elevated reticulocytes (12%), LDH and bilirubin, leading to a diagnosis of acute haemolytic anaemia due to both drugs, which were then stopped.^19^ She received a blood transfusion and recovered with reticulocytes and haemoglobin values back to normal 2 weeks later (Hb 11 g/dL). G6PD testing revealed G6PD deficiency with activity level of 2.5 (4.6 to 13 reference range), and authors highlighted that phenazopyridine, an oxidative drug, was taken for 8 days when it should only be prescribed for 2–3 days.

In 2017 a 46-year-old Italian female in the USA who had been treated for a UTI with trimethoprim/sulfamethoxazole for 5 days with fatigue and anaemia (Hb 11 g/dL) mentioned that she and family members had experienced similar reactions to other medications including NF.^20^ She recovered after discontinuing treatment, and G6PD testing performed afterwards led to a G6PD deficiency diagnosis, which she shared with her family in Sicily leading to five of them also being diagnosed, (mostly males), suggesting they had G6PD Mediterranean variant leading to drug-induced haemolysis.

*Other enzymatic deficiencies*

Haemolytic anaemias have been described related to NF in G6PD normal patients. A 36 year-old woman of British descent was given sulfisoxazole for five days followed by NF for another five days and developed haemolytic anaemia in 1968 in the USA; she was G6PD normal and deficient for erythrocyte glutathione peroxidase (GSH-Px) as measured by enzyme activity in the range of heterozygous adults.^21, 22^ A G6PD normal 41-year-old female patient in the USA recovered after NF, given at 100 mg four times/day for 3 days, was discontinued and she received prednisone and six blood transfusions; the patient (as well as her sister) were shown to be deficient in erythrocyte enolase as reported in 1972.^23^

### Evidence from reviews and other studies

In 1963, a review was submitted at the request of the Study Group of Blood Dyscrasias of the Council on Drugs (AMA) that conducted semi-annual cumulative tabulations of reports since 1957. There were 1962 reports ^24^ in the USA; three patients had received NF alone; no further details were included.

A retrospective study of haemolytic anaemia associated with G6PD deficiency from one centre was published in 1966. G6PD deficiency was determined by a routine laboratory examination of 2874 patients at the Columbia-Presbyterian Medical Centre, New York city (USA) between 1956 and 1964.^25^ 102 patients found G6PD deficient (3.55%), 63 included records for complete analysis. Out of 45 probable drug-induced cases of haemolytic anaemia among these patients, almost half (20) were associated with sulfonamides (sulfadiazine, sulfamerazine, sulfamethazine, and sulfisoxazole) and three were classified as probably due to NF exposure, with another two probably due to NF and sulfa drugs.

In 1967 a study in pregnant women with acute pyelonephritis treated with four different drugs assigned randomly, one of which was NF administered to 87 women, reported haemolytic anaemia and probable hepatitis in two black American patients in the USA who “subsequently showed evidence of G6PD deficiency”.^26^ The maximum daily drug dosage for NF (Furadantin) administered was 540 mg intravenously, then 400 mg orally. Both haemolysis and hepatic reactions subsided in the two patients following drug therapy change and no untoward effects were apparent in either foetus.

In a review of drug-induced haemolysis published in 1967, the authors include as unpublished observations two cases of NF-associated haemolytic anaemia in Sardinia, a region known to have a very high prevalence of G6PD deficiency; from 14 cases of drug-induced haemolytic anaemia in a 500-bed hospital in two years, G6PD deficiency was present in 13, probably including the two cases due to NF.^27^

A study published in 1968 in which NF was administered at 100 mg four times daily for two weeks to 14 individuals to evaluate neurological effects of the drug, reported a G6PD deficient black man among the study subjects, who was described briefly.^28^ He showed elevated methaemoglobin reduction values during baseline studies, with G6PD deficiency confirmed by enzyme assay and was the only study subject showing a fall in haematocrit during NF (from 42% to 36%).

In the discussion section of a report on glutathione stability test results of 323 people in Agra, India published in 1969, the author mentioned as unpublished data two cases of NF-induced haemolysis in the 1966-1968 period without additional details.^29^

In 1969 the results of NF treatment of pregnant patients at an obstetric clinic in South Carolina, USA were reported that included a group of 93 women of whom 73 were black, who received NF 200 or 400 mg daily for 3 or 6 weeks or the remainder of the pregnancy for asymptomatic bacteriuria.^30^ Two patients in this group were diagnosed with G6PD-related anaemia with no haemolysis documented; no further details were included. We did not include these cases to estimate risk as our search terms included “haemolysis” and “haemolytic anaemia” and not all anaemia.

A prospective study published in 1974 on the risk of adverse reactions to antimicrobials at the Shands Teaching Hospital of the University of Florida, USA in patients admitted mid 1969-mid 1972 reported two episodes of haemolytic anaemia in two patients due to NF with no further details provided.^31^

A retrospective study based on review of medical records of 129 G6PD-deficient Kurdish patient records from two medical practices in Israel was published in 1975; all patients’ G6PD enzymatic activity was measured by the Oski and Growney method based on red cell staining with methylene blue.^32^ Possible drug-induced haemolytic episodes were analysed.^33^ There were three pregnant women (19, 33 and 37 years old) who had received NF for UTIs with one of them also receiving streptomycin. The G6PD values obtained in 14 female patients measured by quantitative assay were very low and the authors suggest, considering endogamous marriage in the Kurdish Jewish community at the time, that they may had been homozygotes. These patients were probably all G6PD Mediterranean variant, which is the most prevalent in this population.^34^

A book published in 1977, summarising drug-associated blood dyscrasias, included a chapter on NF.^35^ A total of 42 cases of haemolytic anaemias were reported following NF use, 22 of which occurred in G6PD deficient individuals, all following NF ingestion of 200-800 mg/d for four days to two weeks; haemolysis ceased upon drug withdrawal. Several cases had been reported in the literature including two individuals with hepatotoxicity (Supplementary Table 1 and case report^26^) and others (Supplementary Table 1 and case reports^1, 2, 5, 7, 10, 14, 21, 23^), and two other individuals had received sulfa drugs and another streptomycin^25, 33^).

In 1985, a review of major reactions to NF was published by an independent assessor that was allowed access to databases and information by the developer of NF, Norwich Eaton Pharmaceuticals, on all NF brands from all manufacturers, adverse reaction reports from available publications and regulatory authorities, company studies case reports and other studies since the drug was introduced in 1953, with an estimated 121,430,000 courses of therapy prescribed in the USA.^36^ This review mentions a 1977 review report of 42 cases of haemolytic anaemia due to NF intake (^35^, described above) that included both G6PD deficient and G6PD normal cases in either enolase or glutathione peroxidase deficient patients (see case reports) and patients with no known enzymatic deficiencies.

A report published in 1971 (and mentioned in the above review^36^) on patients treated with different drugs including NF in 1967-1968 at the Massachusetts General Hospital reported one case of haemolytic anaemia among haematological reactions observed for NF out of 757 courses of treatment.^37^

Another review on NF and haemolytic reactions published in 1990 by the Section Head of the Department of Medical Affairs at Norwich Eaton Pharmaceuticals was also based on the company’s adverse reaction database of all possible sources of worldwide data since 1953 that included spontaneous reports from physicians, pharmacists, patients, and company representatives; reports from clinical trials; and reports from published literature.^38^ There were 127 reports of "haemolytic anaemia" or "haemolytic anaemia as a result of G6PD deficiency" "haemolysis," "primaquine-type reaction," and "Mediterranean phenomenon" that included 11 pregnant patients and 9 neonates. Five patients had also received a sulphonamide in addition to NF, which may have therefore contributed to this reaction. While five other patients tested deficient for G6PD, there was no record of G6PD testing for 102 patients, with presumptive or no diagnosis of G6PD deficiency made and little details otherwise available. Of 107 reports, data were available for 65 (61%), of whom 57 (88%) apparently recovered completely. Five patients underwent therapy, one experienced residual morbidity, and two deaths were recorded caused by myocardial infarction and thrombotic thrombocytopenic purpura, respectively, that did not appear to have been related to the haemolytic reaction.

A review from Sweden of 921 reports of adverse reactions to NF received by the Swedish Adverse Drug Reaction Committee in 1966-1976 showed mainly acute pulmonary reactions (43%) and allergic reactions (42%), with a blood dyscrasias group, for which there were no additional details provided, of 20 cases or 2%.^39^ However, two previous reports by a Swedish group in 1973 based on the same database for 1966-1970 showed that among all drug-induced reactions reported (aplastic anaemia, haemolytic anaemia, thrombocytopenia, and agranulocytosis) NF had only resulted in three cases of thrombocytopenia and none of haemolytic anaemia.^40, 41^ A subsequent review published two years later included adverse reactions to NF in Sweden, the UK and the Netherlands in seven different groups, one of which was blood dyscrasias, adding 11 in the UK and six in the Netherlands to the previously reported 20 in Sweden.^42^ The authors, who highlighted the difference between countries in reporting rates, mentioned UTI treatment including NF as long-term follow-up, as one death case in the UK attributed to NF in which the drug had been given for 18 months, as well as NF prescription rates falling in both countries between 1976 and 1979 by 21% (UK) and 39% (Sweden). The recommended NF doses for UTI treatment in Sweden (50 mg three or four times daily for up to two to three weeks) and the UK (100 mg four times daily for two weeks) back then were higher than those currently recommended.

A NF systematic review and meta-analysis published in 2015 that included 27 controlled trials and 4807 patients found low rates of adverse effects that were mild, reversible and predominantly gastrointestinal, and a lack of AEs such as pulmonary fibrosis and hepatotoxicity; the authors commented on what they interpreted as a publication bias focused on NF hypersensitivity reactions.^43^. Citing D’Arcy review (see above^36^), they emphasised very low frequencies estimated for pulmonary reactions (0.001%), hepatic toxicity (0.0003%) neurological events (0.0007%) and haematological events (0.0004%).

Only two deaths were reported in a review, mentioned above and not appearing to have been due to haemolysis,^38^ with no other publication above including deaths attributed to NF-induced haemolytic anaemia.

**References**

1.West M, Zimmerman HJ. Hemolytic anemia in patient receiving nitrofurantoin (furadantin). *J Am Med Assoc* 1956; **162**: 637-9.

2.Kimbro EL, Jr., Sachs MV, Torbert JV. Mechanism of the hemolytic anemia induced by nitrofurantoin (furadantin); further observations on the incidence and significance of primaquine-sensitive red cells. *Bull Johns Hopkins Hosp* 1957; **101**: 245-57.

3.Tarlov AR, Brewer GJ, Carson PE *et al.* Primaquine sensitivity. Glucose-6-phosphate dehydrogenase deficiency: an inborn error of metabolism of medical and biological significance. *Arch Intern Med* 1962; **109**: 209-34.

4.Watson J, Taylor WR, Menard D *et al.* Modelling primaquine-induced haemolysis in G6PD deficiency. *Elife* 2017; **6**.

5.Levy D, Porgess A. Haemolytic anaemia in a child receiving nitrofurantoin. *Arch Dis Child* 1958; **33**: 527-8.

6.Garrett JV, Hallum J, Scott P. Urinary Antiseptics Causing Haemolytic Anaemia in Pregnancy in a West Indian Woman with Red Cell Enzyme Deficiency. *J Obstet Gynaecol Br Commonw* 1963; **70**: 1073-5.

7.Deveber LL, Valentine GH. NITROFURANTOIN AND MEGALOBLASTIC ANAEMIA. *Lancet* 1964; **2**: 697-8.

8.Jeannet M, Perrier CV, Toenz O. [Acute Hemolytic Anemia Caused by Nitrofurantoin in an Iranian Woman Presenting an Erythrocyte Glucose-6-Phosphate-Dehydrogenase Deficiency; Demonstration of the Heterozygosis of the Patient by an Original Method]. *Schweiz Med Wochenschr* 1964; **94**: 939-43.

9.Brewer GJ, Tarlov AR, Alving AS. The methemoglobin reduction test for primaquine-type sensitivity of erythrocytes. A simplified procedure for detecting a specific hypersusceptibility to drug hemolysis. *JAMA* 1962; **180**: 386-8.

10.Pritchard JA, Scott DE, Mason RA. Severe anemia with hemolysis and megaloblastic erythropoiesis. A reaction to nitrofurantoin administered during pregnancy. *JAMA* 1965; **194**: 457-9.

11.Vergnes H, Clerc A. Erythrocyte Enzyme Activity in Pregnancy *Lancet* 1968; **292**: 834.

12.Maszkiewicz W, Soltys R. [A case of drug-induced hemolytic anemia with methemoglobinemia in a newborn]. *Wiad Lek* 1969; **22**: 1793-6.

13.Brewer GJ, Tarlov AR. Studies on the mechanism of primaquine-type hemolysis: The effect of methylene blue. *Clin Res* 1961; **9**: 65 (abstract).

14.Carpel EF. Hemolysis induced by nitrofurantoin. *Pa Med* 1970; **73**: 49-50.

15.Lavelle KJ, Atkinson KF, Kleit SA. Hyperlactatemia and hemolysis in G6PD deficiency after nitrofurantoin ingestion. *Am J Med Sci* 1976; **272**: 201-4.

16.Bruel H, Guillemant V, Saladin-Thiron C *et al.* [Hemolytic anemia in a newborn after maternal treatment with nitrofurantoin at the end of pregnancy]. *Arch Pediatr* 2000; **7**: 745-7.

17.Ghimire KB, Nepal B. Dyspnea after treatment of recurrent urinary tract infection. *Cleve Clin J Med* 2013; **80**: 690-5.

18.van de Mheen L, Smits SM, Terpstra WE *et al*. Haemolytic anaemia after nitrofurantoin treatment in a pregnant woman with G6PD deficiency. *BMJ Case Rep* 2014; **2014**.

19.Nasir I. Acute anemia after a urinary tract infection (UTI): A case of hemolytic anemia in a newly diagnosed glucose-6-phosphate dehydrogenase (G6PD) deficient patient (an abstract from the 37th Annual Meeting of the Society of General Internal Medicine, 2014, San Diego, CA, USA) *Journal of general internal medicine* 2014; **29 Suppl 1**: S295.

20.Sheh T, Tsai I. G6PD Deficiency and a Family’s Myth of Medication Allergies. *Proceedings of UCLA Healthcare (Clinical Vignette)* 2017; **20**.

21.Steinberg M, Brauer MJ, Necheles TF. Acute hemolytic anemia associated with erythrocyte glutathione-peroxidase deficiency. *Arch Intern Med* 1970; **125**: 302-3.

22.Steinberg MH, Necheles TF. Erythrocyte glutathione peroxidase deficiency. Biochemical studies on the mechanisms of drug-induced hemolysis. *Am J Med* 1971; **50**: 542-6.

23.Stefanini M. Chronic hemolytic anemia associated with erythrocyte enolase deficiency exacerbated by ingestion of nitrofurantoin. *Am J Clin Pathol* 1972; **58**: 408-14.

24.Best WR. Drug-associated blood dyscrasias. Recent additions to the Registry. *Jama* 1963; **185**: 286-90.

25.Burka ER, Weaver Z, 3rd, Marks PA. Clinical spectrum of hemolytic anemia associated with glucose-6-phosphate dehydrogenase deficiency. *Ann Intern Med* 1966; **64**: 817-25.

26.Hibbard L, Thrupp L, Summeril S *et al.* Treatment of pyelonephritis in pregnancy. *Am J Obstet Gynecol* 1967; **98**: 609-15.

27.Dausset J, Contu L. Drug-induced hemolysis. *Annu Rev Med* 1967; **18**: 55-70.

28.Toole JF, Gergen JA, Hayes DM *et al.* Neural Effects of Nitrofurantoin. *Archives of Neurology* 1968; **18**: 680-7.

29.Mital VP. Glutathione stability of red cells--observations on a sample from Agra. *Indian J Med Sci* 1969; **23**: 483-7.

30.House TE, Williams BL, Jr., Meares GM *et al.* Pregnancy complicated by urinary tract infections. *Obstet Gynecol* 1969; **34**: 670-4.

31.Caldwell JR, Cluff LE. Adverse reactions to antimicrobial agents. *Jama* 1974; **230**: 77-80.

32.Oski FA, Growney PM. A Simple Micromethod for the Detection of Erythrocyte Glucose-6-Phosphate Dehydrogenase Deficiency. *J Pediatr* 1965; **66**: 90-3.

33.Herman J, Ben-Meir S. Overt hemolysis in patients with glucose-6-phosphate dehydrogenase deficiency: a survey in general practice. *Isr J Med Sci* 1975; **11**: 340-6.

34.Oppenheim A, Jury CL, Rund D *et al.* G6PD Mediterranean accounts for the high prevalence of G6PD deficiency in Kurdish Jews. *Hum Genet* 1993; **91**: 293-4.

35.Swanson M, Cook R. Nitrofurantoin. *Drugs, Chemicals, and Blood Dyscrasias: A Summary of Blood Abnormalities Associated with Exposure to Specific Drugs and Chemicals*. Hamilton, Illionois, USA: Drug Intelligence Publications, 1977; 684-395.

36.D'Arcy PF. Nitrofurantoin. *Drug Intell Clin Pharm* 1985; **19**: 540-7.

37.Koch-Weser J, Sidel VW, Dexter M *et al.* Adverse reactions to sulfisoxazole, sulfamethoxazole, and nitrofurantoin. Manifestations and specific reaction rates during 2,118 courses of therapy. *Arch Intern Med* 1971; **128**: 399-404.

38.Gait JE. Hemolytic reactions to nitrofurantoin in patients with glucose-6-phosphate dehydrogenase deficiency: theory and practice. *DICP* 1990; **24**: 1210-3.

39.Holmberg L, Boman G, Bottiger LE *et al.* Adverse reactions to nitrofurantoin. Analysis of 921 reports. *Am J Med* 1980; **69**: 733-8.

40.Böttiger LE, Westerholm B. Acquired haemolytic anaemia. I. Incidence and aetiology. *Acta Med Scand* 1973; **193**: 223-6.

41.Bottiger LE, Westerholm B. Drug-induced blood dyscrasias in Sweden. *Br Med J* 1973; **3**: 339-43.

42.Penn RG, Griffin JP. Adverse reactions to nitrofurantoin in the United Kingdom, Sweden, and Holland. *Br Med J (Clin Res Ed)* 1982; **284**: 1440-2.

43.Huttner A, Verhaegh EM, Harbarth S *et al.* Nitrofurantoin revisited: a systematic review and meta-analysis of controlled trials. *J Antimicrob Chemother* 2015; **70**: 2456-64.
